# Unveiling the Molecular Landscape of MPO in Kikuchi's Disease: Protein Expression, mRNA Levels, and Genetic Polymorphisms

**DOI:** 10.1111/ijlh.14509

**Published:** 2025-07-19

**Authors:** Chen Chang, MengNa Zhang, Yu Chang, Yu Ma, Wensheng Li

**Affiliations:** ^1^ Department of Pathology Shaanxi Provincial People's Hospital Xi'an China; ^2^ Department of Pathology Affiliated Hospital of Shaanxi University of Chinese Medicine Xianyang China; ^3^ Institute of Medical Research, Northwestern Polytechnical University Xi'an China

**Keywords:** gene polymorphism, Kikuchi's disease, *MPO* mRNA, myeloperoxidase

## Abstract

**Purpose:**

This study examines the protein expression and mRNA levels of myeloperoxidase (MPO) in patients with Kikuchi's disease, and explores the association between the MPO‐463G/A polymorphism and Kikuchi's disease.

**Methods:**

Paraffin blocks from 43 patients with Kikuchi's disease were collected, and paraffin blocks from patients with reactive hyperplastic lymph nodes, granulomatous inflammation, and myeloid sarcoma were used as controls. Immunohistochemistry (IHC), quantitative reverse transcription polymerase chain Reaction (qRT‐PCR) and Sanger sequencing were used to detect the relevant variants.

**Results:**

The positive rate of MPO protein expression in the Kikuchi's disease group was 100% by IHC. In comparison to the control group, patients with Kikuchi's disease exhibited elevated *MPO* mRNA expression levels, which demonstrated a positive correlation with protein expression levels. Kikuchi's disease and reactive hyperplastic lymph nodes displayed distinct genotypes at the MPO‐463 locus, with mutation phenotypes of 7% and 30%, respectively. The G allele at this locus emerged as a risk factor.

**Conclusions:**

In Kikuchi's disease, both the protein and mRNA expression levels of MPO are elevated, and the high expression of mRNA is positively correlated with the protein expression levels. The polymorphism at the MPO‐463 locus may be associated with the occurrence of Kikuchi's disease.

## Graphical abstract

This study investigates myeloperoxidase (MPO) in Kikuchi's disease, analyzing protein expression and mRNA levels in 43 patients. Results reveal 100% MPO protein positivity, elevated mRNA expression, and a positive correlation between mRNA and protein levels. The MPO‐463 locus shows distinct genotypes in Kikuchi's disease and controls, suggesting a potential risk association. This study highlights heightened MPO expression and genetic factors in Kikuchi's disease.

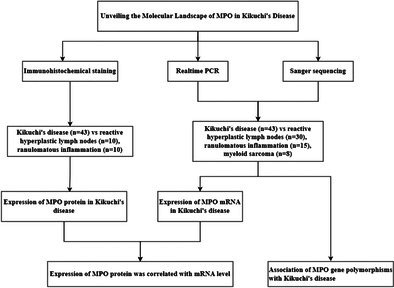

## 1 Introduction

Kikuchi's disease, also known as histiocytic necrotizing lymphadenitis (HNL), is a benign, self‐limiting disease that affects young women, with a high incidence in Asia and an increasing trend in recent years. The etiology and pathogenesis of Kikuchi's disease are unknown, but some studies suggest it is associated with viral infection or autoimmune disease [1]. Histologically, Kikuchi‐Fujimoto disease is characterized by paracortical expansion of lymph nodes with patchy necrosis, abundant karyorrhectic debris, and a notable absence of neutrophils and eosinophils. It exhibits three evolving histologic patterns: proliferative, necrotizing, and xanthomatous, as initially described by Kuo and reaffirmed by subsequent reviews [2, 3].

Myeloperoxidase (MPO) serves as a highly sensitive and specific marker in myeloid cells and myeloid tumors. Recent investigations have substantiated the heightened expression of MPO in Kikuchi's disease, establishing it as a characteristic marker for the disease's diagnosis. Positive MPO expression in the cytoplasm of CD68‐positive histiocytes in Kikuchi disease has been observed in some studies [4, 5, 6], indicating the potential for proliferating histiocytes in Kikuchi disease to co‐express CD68 and MPO. Moreover, certain studies have reported that plasmacytic dendritic cells expressing CD123 also exhibit MPO expression [7]. Nevertheless, limited research has delved into the characteristics and expression patterns of positive MPO expression in Kikuchi's disease.

The current assessment of MPO expression in Kikuchi disease is limited to the detection of protein expression levels using IHC. Discrepancies between mRNA and protein expression may arise due to post‐transcriptional regulation and post‐translational modifications. Additionally, gene polymorphisms may influence the transcription and translation processes, thereby affecting an organism's susceptibility to disease. Notably, investigations into MPO gene polymorphisms have predominantly centered around G/A mutations at nucleotide 463 of the promoter region [8, 9, 10]. Numerous studies have indicated that the 463G/A polymorphism is associated with susceptibility to various conditions, including lung cancer, acute promyelocytic leukemia, and coronary artery disease [11, 12, 13, 14].

In this study, immunohistochemical staining will be conducted on paraffin blocks obtained from individuals with Kikuchi's disease and controls. This aims to summarize the clinical, pathological, and immunophenotypic characteristics of Kikuchi's disease. The investigation will include the detection of *MPO* mRNA expression to explore the correlation between its protein and mRNA expression levels. Additionally, we will examine the relationship between the MPO‐463G/A polymorphism and the susceptibility of the Kikuchi disease population.

## 2 Patients and Methods

### 2.1 The Study Participants

Sixty‐four paraffin block samples diagnosed with Kikuchi's disease from 01/07/2012 to 31/08/2021 were collected from the Department of Pathology at Shaanxi Provincial People's Hospital. After reviewing pathological sections and clinical data, and excluding unqualified paraffin blocks and cases with incomplete data, we selected 43 cases of Kikuchi's disease with precise diagnoses, complete paraffin blocks, and comprehensive clinical data for inclusion in this study. Histological classification of Kikuchi's disease into proliferative (PT), necrotizing (NT), and xanthomatous (XT) subtypes was performed according to established morphological criteria. After reviewing pathological sections and clinical data, and excluding unqualified paraffin blocks and cases with incomplete data, we selected 43 cases of Kikuchi's disease with definitive diagnoses and complete clinical records and tissue materials. These 43 paraffin blocks were used consistently across all experimental procedures, including immunohistochemistry (IHC), quantitative reverse transcription PCR (qRT‐PCR), and Sanger sequencing. For IHC, we used paraffin blocks from 10 patients with reactive hyperplastic lymph nodes and 10 with granulomatous inflammation as controls. For qRT‐PCR and Sanger sequencing, the control group included 30 patients with reactive hyperplastic lymph nodes, 15 with granulomatous inflammation, and 8 with myeloid sarcoma. Among these, 10 reactive hyperplasia cases overlapped with the IHC control group, while 20 were newly added for expanded analysis. This ensured consistency within the Kikuchi's disease group and statistical adequacy in control comparisons. All cases were confirmed by two attending pathologists upon reviewing the sections.

During or after the data collection, we did not obtain any information that could identify individual participants. This study was approved by the Ethics Committee of the Shaanxi Provincial People's Hospital (No. 2023R057)and was conducted in accordance with the Helsinki Declaration. Since this study is a retrospective study and all patient data are anonymous, informed consent was waived with the approval of the Ethics Committee of the Shaanxi Provincial People's Hospital.

### 2.2 HE and Immunohistochemical Staining

All specimens were adequately fixed in neutral formaldehyde, then dehydrated, paraffin‐embedded, and sectioned at a thickness of 3–4 μm. Subsequently, they were deparaffinized for HE staining and Max Vision immunohistochemical staining, respectively. The procedure included endogenous peroxidase blocking, primary antibody incubation (CD3, CD5, CD43 and MPO is ready‐to‐use), secondary antibody incubation, DAB staining, hematoxylin staining, PBS bluing, and final blocking.

### 2.3 Determination of Immunohistochemical Results

MPO‐positive staining is observed in the cytoplasm. The interpretation of MPO staining results involves scoring based on the percentage of colored cells in the field of view: 0 for no positive cells, 1 for ≤ 25%, 2 for 26%–50%, 3 for 51%–75%, and 4 for > 75%. Additionally, points are allocated according to the intensity of staining: 0 for no staining, 1 for weak staining, 2 for moderate intensity staining, and 3 for intense staining. These two scores are then multiplied, and a product of ≥ 1 is considered positive. Importantly, areas of overt necrosis were excluded from scoring due to potential degradation of antigenicity. Only viable surrounding tissue was used for semi‐quantitative evaluation. Specifically, a score of 0 is interpreted as negative (−), 1–4 as weakly positive (+), 5–8 as moderately positive (++), and 9–12 as strongly positive (+++).

### 2.4 Realtime PCR and Sanger Sequencing

After stable fixation and embedding of the specimen, five 8 μm thick paraffin rolls were collected and placed in a 1.5 mL EP tube. RNA extraction was performed using the nucleic acid extraction kit (OMEGA). A 20 μL system was configured, and 4 μL of 5X PrimeScript RT Master Mix solution was added to a 0.2 mL centrifuge tube. Next, 1000 ng of RNA sample was added, and enzyme‐free water was added to a total of 20 μL. After thorough mixing, the reverse transcription reaction was carried out under the conditions set at 37°C for 15 min and 85°C for 5 s, followed by transfer to 4°C.

Primer design was performed using Primer 5.0 software, and the MPO gene was transcribed using NM_000250.2, and the primer sequences are shown in Table S1. The qRT‐PCR amplification reaction system was set at 20 μL. Three replicate wells were prepared for each sample, and the reaction mixture was assembled as outlined in Table S2. Detection was performed using a Roche 480 analyzer, and the software cycle program was set as follows: Phase I, 95°C for 30 s (1 cycle); Phase II, 95°C for 5 s, 62°C for 1 min, 72°C for 30 s (45 cycles). The resulting products were sent to Shanghai Biotech for sequencing, and the sequence results were compared and analyzed using BLAST on NCBI.

### 2.5 Statistical Analysis

The SPSS 25.0 software package was employed for statistical analysis. All measurement data were expressed as mean ± standard deviation (x ± s). The t‐test was used for comparisons between two groups, while one‐way ANOVA was utilized for comparisons between multiple groups. Spearman's correlation coefficient analysis was performed for correlation analysis, direct counts for gene frequencies, and Fisher's exact probability method was employed to test whether the genotype frequencies of each group satisfied Hardy–Weinberg equilibrium. Differences in genotype and allele frequencies between groups were statistically treated using Fisher's exact probability method. The effect of genetic polymorphism on the risk of Kikuchi's disease was statistically measured by the odds ratio (OR) and its 95% confidence interval (CI). *p* < 0.05 was considered statistically significant.

## 3 Results

### 3.1 Clinical Features of Kikuchi's Disease

Of the 43 cases of Kikuchi's disease, 16 were male and 27 female, with a male‐to‐female ratio of 0.59:1, aged 13–64 years, with a mean age of 32 years and a median age of 28 years. Thirty‐three patients had clinical signs of persistent fever and lymph node enlargement. Sixteen cases had a temperature above 39°C and 17 cases had a temperature between 37.5°C and 39°C. The duration of fever ranged from 4 days to 2 months, with a mean duration of 16.5 days. The sites of occurrence in this group of cases included bilateral cervical lymph nodes in 4 cases, unilateral cervical lymph nodes in 31 cases, supraclavicular lymph nodes in 3 cases, submandibular lymph nodes in 2 cases, and axillary lymph nodes in 3 cases.

### 3.2 Histopathological Morphological Features

**FIGURE 1:**
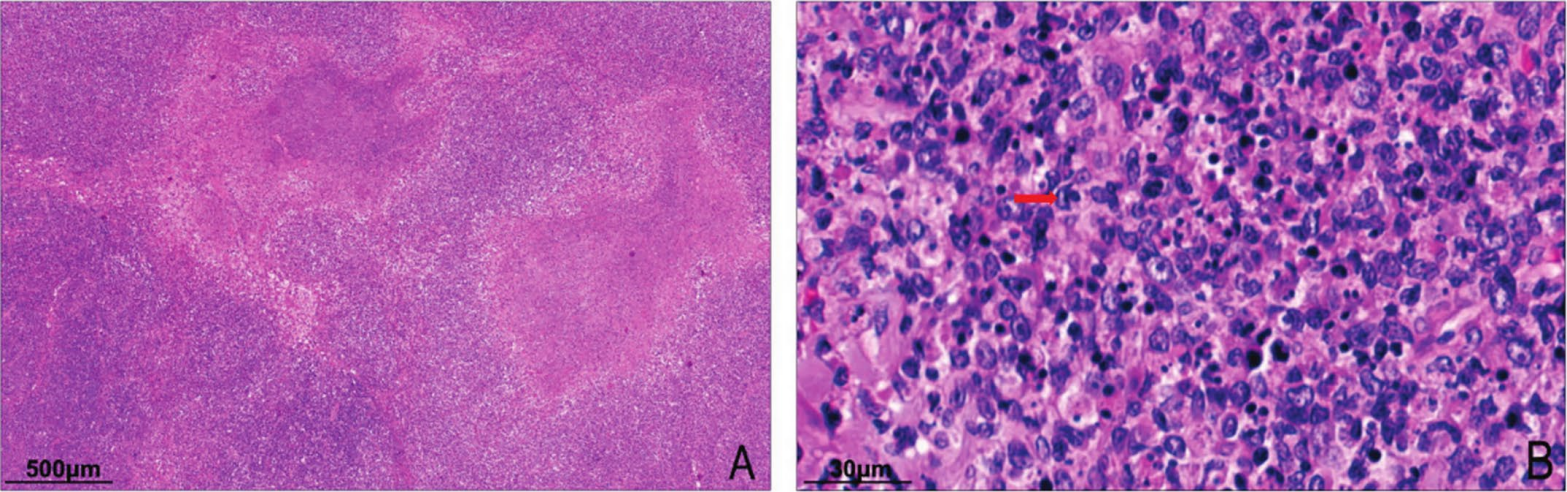
Morphological features of Kikuchi's disease (HE). (A) Disturbed lymph node structure with irregular foci of necrosis. (B) The lesion area shows numerous histiocytes (red arrows), many of which contain phagocytosed nuclear debris, accompanied by surrounding lymphocytes. A variable number of apoptotic cells and karyorrhectic debris are also observed.

In Kikuchi's disease, the lymph nodes are structurally disorganized, with irregularly mapped lesions dominated by crescent‐shaped histiocytes that phagocytose nuclear debris, non‐phagocytic histiocytes, immunoblasts, atypical T lymphocytes, and plasmacytic dendritic cells (Figure 1), but neutrophils and plasma cells are absent. Each case saw a variable number of apoptotic cells or nuclear debris and coagulative necrosis foci; 75% of the 43 cases of Kikuchi disease had both PT and NT type changes, with NT type predominating in 53.5% (23/43), PT type in 39.5% (17/43) and XT type in 7.0% (3/43).

### 3.3 T‐Cell Antigen Expression and CD5 Loss in Kikuchi's Disease

**FIGURE 2:**
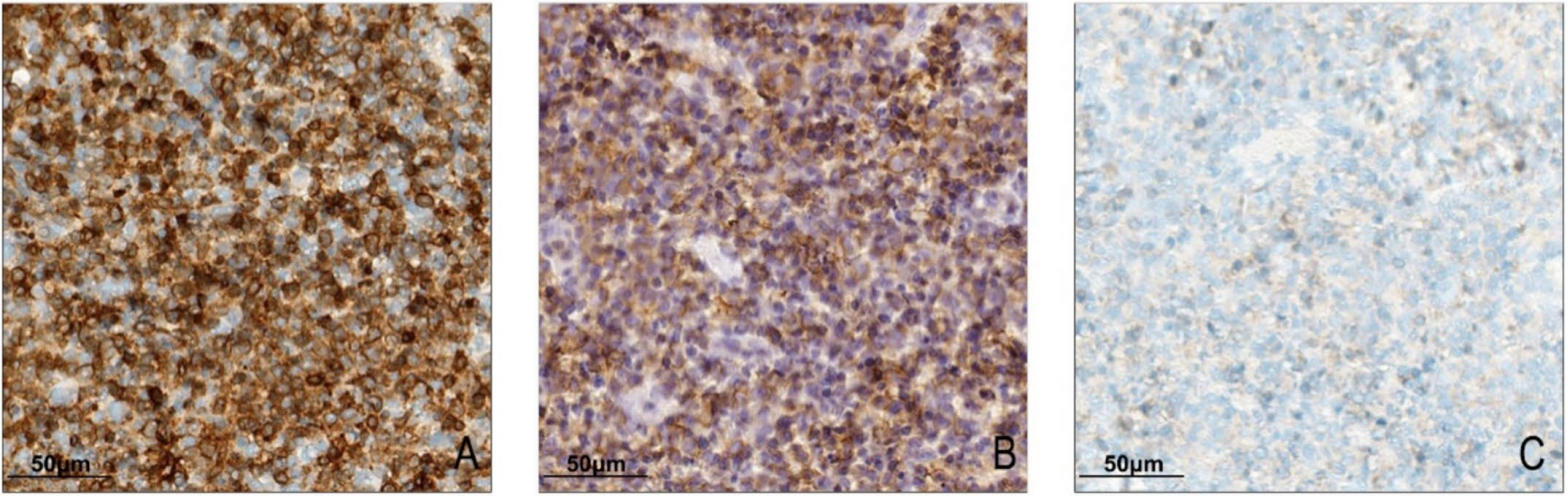
Immunohistochemical staining of T‐cell markers in Kikuchi's disease. (A) CD3 shows diffuse positive staining in lymphoid cells. (B) CD43 is diffusely expressed in lymphoid cells. (C) CD5 expression is nearly absent, indicating complete antigen loss.

Immunohistochemical analysis of T‐cell markers was performed in the Kikuchi disease group and reactive hyperplastic lymph node controls. CD5 antigen loss was observed exclusively in Kikuchi disease cases, with 67.4% (29/43) of cases showing partial or complete loss. Complete CD5 negativity was identified in 4.7% (2/43) of cases (Figure 2). In most cases with CD5 loss, apoptotic cells and nuclear debris were observed in the affected areas. In contrast, CD3 and CD43 expression remained intact across all Kikuchi disease samples, with no evidence of antigen loss. None of the reactive hyperplastic lymph node controls exhibited antigen loss for CD3, CD5, or CD43. The difference in T‐cell antigen loss between Kikuchi disease and reactive hyperplasia was statistically significant (*p* < 0.001, Table S3). The rate of CD5 antigen loss varied among the histologic subtypes: 41.2% (7/17) in the proliferative type (PT), 87.0% (20/23) in the necrotizing type (NT), and 66.7% (2/3) in the xanthomatous type (XT). The frequency of CD5 loss was significantly associated with histologic subtype (*p* = 0.005, Table S4), occurring more frequently in NT and XT types.

### 3.4 Expression of MPO Protein in Kikuchi Disease

**FIGURE 3:**
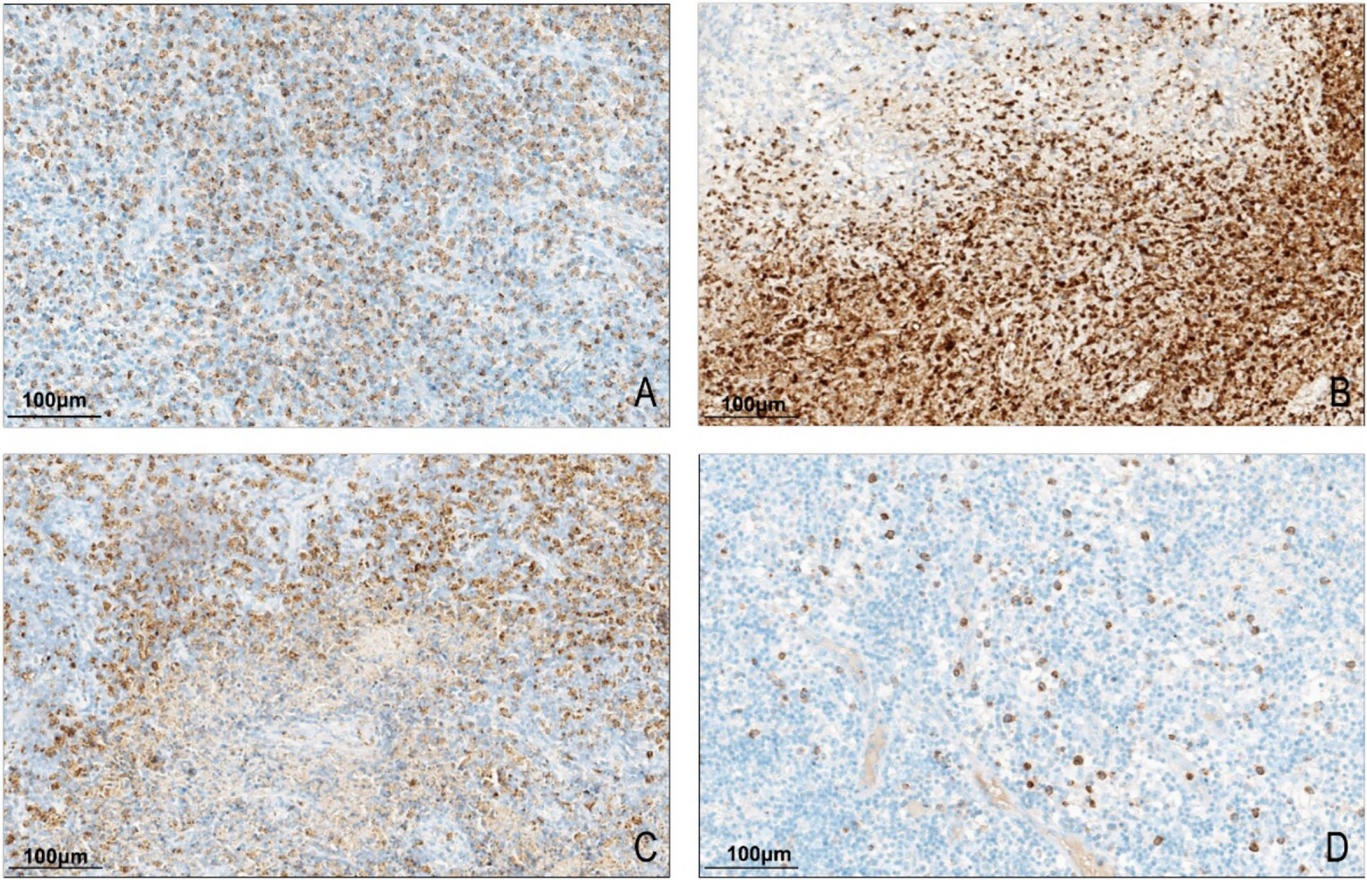
Immunohistochemical staining for MPO in Kikuchi disease. (A) MPO is diffusely positive throughout the lesion area. (B) MPO is strongly positive in the necrotic area and weakly positive or scattered positive in other surrounding lesion areas. (C) MPO is scattered or weakly expressed in the necrotic area and diffusely positive in the surrounding area. (D) MPO is scattered throughout the lesion area.

MPO positivity was localized in the cytoplasm and appeared as granules of varying sizes. All 43 cases of Kikuchi disease expressed MPO, of which 23.3% (10/43) were weakly positive, 37.2% (16/43) were moderately positive, and 39.5% (17/43) were strongly positive. MPO expression patterns in Kikuchi's disease were fourfold: (i) the entire lesion was diffusely positive, uniformly, 58.1% (25/43) (Figure 3A); (ii) the necrotic area was strongly positive, and the other surrounding lesions were weakly positive or scattered positive, 9.3% (4/43) (Figure 3B); (iii) the necrotic area was weakly expressed, and the other lesions were diffusely positive, 16.3% (7/43) (Figure 3C); (iv) the number of positive cells in the entire lesion was low and scattered, 16.3% (7/43) (Figure 3D). While some necrotic areas displayed MPO positivity, these patterns were documented qualitatively to describe distribution features and were not included in the scoring process. However, in the control group, both reactive hyperplastic lymph nodes and granulomatous inflammatory MPO‐positive cells were neutrophils with a scattered distribution, significantly different from Kikuchi's disease (*p* < 0.001, Table S5).

### 3.5 Expression of MPO mRNA in Kikuchi Disease

**FIGURE 4:**
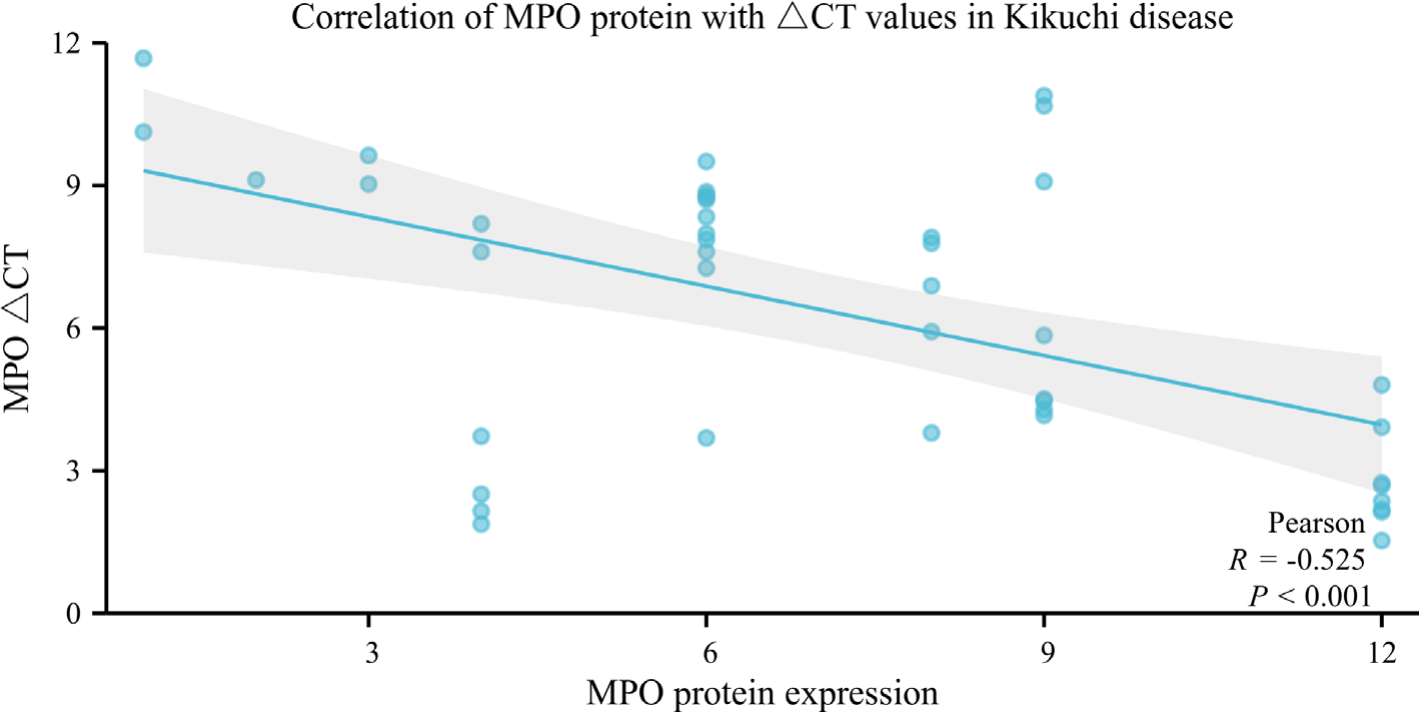
Correlation of MPO protein with *MPO* ΔCt values in Kikuchi disease.

The mRNA expression of MPO was specific to Kikuchi's disease and its controls. Its expression was significantly higher in Kikuchi's disease compared to the reactive hyperplastic lymph node group and the granulomatous inflammatory group, with statistically significant differences. In contrast, there were no differences between them and the myeloid sarcoma group (Table S6). Correlation analysis between MPO mRNA expression levels and histologic subtype showed no statistically significant differences among PT, NT, and XT groups (*p* = 0.235, Table S7). The correlation of its expression with clinical features showed no statistically significant association between *MPO* mRNA expression levels and gender, age, lymph node size, and body temperature in Kikuchi's disease (*p* > 0.05). MPO ∆Ct values were negatively correlated with MPO protein expression in Kikuchi's disease (*r* = −0.525, *p* < 0.001) (Figure 4, Table S8), indicating a positive correlation between MPO protein and mRNA expression levels in Kikuchi's disease.

### 3.6 Association of MPO Gene Polymorphisms With Kikuchi Disease

A total of 73 cases in the Kikuchi disease and control groups were sequenced, and mutations were identified exclusively at the MPO‐463 locus (Figure S1), with the presence of mutant genotypes GA and AA. Among the 43 cases of Kikuchi disease, 93.0% were GG, 4.7% were GA, and 2.3% were AA, resulting in a mutation rate of 7.0%. In the 30 cases of reactive hyperplastic lymph nodes, 70% were GG, 26.7% were GA, and 3.3% were AA, resulting in a mutation rate of 30%. The mutation rates between these two groups were statistically significantly different (*p* = 0.015) (Table S9). The Hardy–Weinberg law of genetic equilibrium was applied to test both the Kikuchi disease group and the reactive hyperplastic lymph node group, and the results of both groups (*p* > 0.05) satisfied the Hardy–Weinberg law, indicating that the data from both groups are representative of the population (Table S10).

Furthermore, the MPO‐463 G/A locus allele frequency distribution differed between the two groups (*p* = 0.022). Analysis revealed that the risk of Kikuchi disease was 0.175 times higher with the GA and AA genotype frequencies than with the GG genotype, suggesting that carrying the GA and AA genotypes may be associated with a lower likelihood of developing Kikuchi disease. Additionally, the risk of Kikuchi disease was 0.244 times higher with the A allele frequency than with the G allele frequency, indicating that carrying the A allele reduces the risk of the disease, indicating a potential protective trend (Table 1). No statistically significant difference was observed between the MPO‐463 G/A locus polymorphism and MPO mRNA expression levels (Table S11).

**TABLE 1:** Association between MPO‐463 G/A gene polymorphisms and susceptibility to Kikuchi disease.

Genotype	Kikuchi disease (*n*, %)	Reactive hyperplastic lymph nodes (*n*, %)	OR (95% CI)	*p*
GG	40 (93.0)	21 (70)	1	0.015
GA+AA	3 (7.0)	9 (30)	0.175 (0.043–0.716)	
G	82 (95.3)	50 (83.3)	1	0.022
A	4 (4.7)	10 (16.7)	0.244 (0.073–0.819)	

*Note: p* < 0.05 is statistically significant.

## 4 Discussion

The MPO gene, located in the q23 region of the long arm of chromosome 17, comprises 12 exons and is approximately 14 638 bp long [15]. It encodes a hemoglobinase with a hemoglobin cofactor, produced by neutrophils and monocytes. Acting as a key player in killing microorganisms within phagocytes, MPO serves as a highly sensitive and specific biomarker for myeloid cells and myeloid tumors [16]. While it was initially a significant marker for diagnosing acute myeloid leukemia, subsequent research revealed its expression also in other hematopoietic disorders such as Kikuchi's disease. Several studies have attempted to explain the presence of positive MPO expression in Kikuchi's disease despite the lack of neutrophils. While earlier hypotheses suggested MPO may derive from neutrophil degranulation stimulated by nuclear debris, this is unlikely in Kikuchi's disease due to the absence of neutrophils in the lesion. A more plausible explanation is that circulating monocytes expressing MPO may retain or re‐express it after migrating into lymph nodes and differentiating into histiocytes. This has been supported by recent immunophenotypic analyses showing MPO^+^/CD68^+^ histiocyte populations in affected tissues [5]. Additionally, it has been suggested that CD68 and MPO‐positive monocytes in peripheral blood may be attracted to lymph nodes to function as granulocytes, providing the MPO required for inflammatory and cell death mechanisms in this specific case [17].

Although MPO is expressed explicitly in Kikuchi's disease, its expression pattern has yet to be fully reported. In our study, all 43 cases of Kikuchi disease exhibited MPO expression. Four positive expression patterns were identified: 58.1% showed uniform diffuse positivity throughout the lesion area; 9.3% displayed strong positivity in the necrotic area with weak or scattered positivity in the surrounding area; 16.3% exhibited weak positivity in the necrotic area with diffuse positivity in the surrounding area; and 16.3% had a low number of positive cells with scattered distribution. In combination with morphology, MPO expression within histiocytes is an unusual finding in benign lymphadenopathies and has been reported as a characteristic immunophenotypic feature of Kikuchi's disease. While not entirely disease‐specific, this pattern may help distinguish it from other reactive conditions [4, 7].

In addition to MPO expression, we analyzed the expression of T‐cell–associated markers to further characterize the immune background. CD3 and CD43 were diffusely expressed, confirming T‐cell predominance. CD5 antigen loss was observed in 67.4% of Kikuchi disease cases and was significantly associated with the necrotizing and xanthomatous subtypes. Loss of CD5 expression has been previously associated with immune dysregulation and apoptosis in T‐cell populations, suggesting that its absence may reflect heightened immune activation and tissue injury severity in these cases. Although CD68 staining was not performed in this study, previous reports have demonstrated that CD68‐positive histiocytes in Kikuchi's disease can exhibit cytoplasmic MPO expression [4, 5, 6], supporting our interpretation of MPO‐positive cells as histiocytes. We acknowledge this as a limitation and plan to include CD68 and other histiocytic markers in future prospective studies.

Kikuchi's disease exhibits a spectrum of histopathologic patterns that may represent different phases of disease progression. According to Perry and Choi (2018), three major histologic subtypes have been described: PT, NT, and XT, each reflecting distinct morphologic features and immune activity [3]. In our study, these subtypes were identified and their correlations with immunohistochemical features were analyzed. Notably, CD5 antigen loss was most frequent in NT and XT types, while least common in PT, suggesting that CD5 downregulation may be associated with the extent of tissue injury and apoptosis in advanced stages of the disease. Furthermore, MPO‐positive histiocytes were observed across all subtypes, supporting their role in the pathogenesis regardless of morphologic stage. These findings are consistent with the evolving‐phase model proposed in the literature and further emphasize the importance of histologic subclassification in interpreting immunophenotypic and molecular features of Kikuchi's disease.

While positive expression of MPO protein has been detected in Kikuchi's disease, whether the protein is MPO produced by myeloid cells or structurally similar to its protein remains unclear. Therefore, in this study, *MPO* mRNA expression in Kikuchi's disease was measured using qPCR, confirming that the positive MPO protein is indeed the MPO produced by myeloid cells. *MPO* mRNA levels in the control group with granulomatous inflammation, which also included many histiocytes, were deficient and significantly different compared to the Kikuchi disease group (*p* < 0.05). This suggests that *MPO* mRNA expression by histiocytes is specifically present in Kikuchi's disease. There was no significant difference in MPO mRNA expression between the Kikuchi disease group and the myeloid sarcoma group, indicating that the MPO transcript levels in Kikuchi's disease are within a similar range to those observed in MPO‐rich myeloid neoplasms. Therefore, we speculate on a possible mechanism for MPO expression in Kikuchi's disease: monocytes from peripheral blood enter the diseased lymph nodes, and MPO does not disappear after developing into histiocytes but continues to function. This phenomenon may be related to the microenvironment in the lymph nodes of Kikuchi's disease. In this study, we found that mRNA expression of MPO was positively correlated with protein expression levels in Kikuchi's disease, with protein expression levels increasing as transcript levels increased. In our study, the level of MPO mRNA expression in Kikuchi's disease was found to be comparable to that observed in myeloid sarcoma. It is important to emphasize that this comparison reflects the quantitative range of transcript expression, not the cellular origin or pathogenesis. In myeloid sarcoma, MPO is expressed by malignant cells of myeloid lineage, often with neutrophilic differentiation. In contrast, MPO expression in Kikuchi's disease likely originates from a subset of histiocytes with retained or aberrantly activated myeloid transcriptional programs. This similarity in expression levels may reflect an immune activation profile, rather than direct cellular equivalence.

Genetic polymorphisms play a crucial role in unveiling human tolerance and susceptibility to diseases and toxicants, the diversity of clinical disease manifestations, and the study and clinical diagnosis of hereditary conditions. The close relationship between MPO‐463 locus G/A polymorphisms and various diseases has been gradually recognized. Previous studies have demonstrated that MPO can contribute to disease through different pathways, while its genetic polymorphisms can reduce susceptibility to specific pathologies, thereby exerting a protective effect on the body [18]. The genotype distributions of MPO‐463G/A in both the Kikuchi disease group and the reactive hyperplastic lymph node group conformed to Hardy–Weinberg equilibrium (*p* > 0.05), indicating that both groups were genetically representative and suitable for association analysis. In this experiment, mutations were identified at the MPO‐463 locus through Sanger sequencing. The mutation rate was notably high at 30% in the reactive hyperplastic lymph node group, significantly different from the 7.0% in the Kikuchi disease group. The mutant genotypes were GA and AA, consistent with literature findings [11, 19]. Previous studies have reported a stable polymorphic mutation rate of approximately 25.5%–29.1% for the GA and AA genotypes in healthy Asian populations at this locus [20, 21, 22]. This rate is close to the 30% mutation rate in the reactive proliferative lymph node group in this paper, indicating the reliability of the data in this paper. The reactive hyperplastic lymph nodes selected for this control group mainly comprised paracolic lymph nodes of patients with cholecystitis without other underlying diseases, making this group representative of the healthy population. The statistically significant difference in the distribution of allele frequencies at the MPO‐463G/A locus between the two groups suggests a correlation between this locus and susceptibility to Kikuchi disease. The GA and AA genotypes were associated with a reduced risk of developing Kikuchi disease compared to the GG genotype. Comparing the A allele frequency with the G allele frequency, the risk of developing Kikuchi disease was approximately 0.244 times greater for those carrying the A allele than the G allele frequency. This implies that the A allele at the MPO‐463G/A locus may be associated with a lower risk of Kikuchi disease, while the G allele may be associated with an increased risk. It has been reported that the G‐A variant disrupts the binding site of the transcription factor SP1 and that the G allele is 25 times more capable of transcribing MPO than the A allele, resulting in a significant variation in mRNA expression levels and affecting MPO activity [23]. However, the results of this experiment showed no significant difference between the MPO‐463G/A locus gene polymorphism and MPO mRNA expression level. This discrepancy may be attributed to the small sample size of this experiment or influenced by the source of the study population, ethnicity, and region.

## 5 Conclusion

In summary, the research explores the intricate characteristics of the MPO gene in Kikuchi's disease. The study delves into the distinct expression patterns of MPO in Kikuchi's disease, particularly within histiocytes, providing novel insights into its potential involvement in the disease's microenvironment. Furthermore, the investigation uncovers a correlation between MPO‐463 locus G/A polymorphisms and Kikuchi's disease, shedding light on genetic factors influencing disease risk. Despite revealing mutations at the MPO‐463 locus and their potential impact on disease susceptibility, the study finds no significant correlation between MPO‐463G/A polymorphisms and MPO mRNA expression levels, suggesting complex interactions that warrant further exploration.

## Author Contributions

Conceptualization: Wensheng Li. Data curation: Mengna Zhang. Formal analysis: Chen Chang. Investigation: Yu Chang. Methodology: Yu Ma. Resources: Wensheng Li. Supervision: Yu Ma. Writing – original draft: Chen Chang. Writing – review and editing: Chen Chang and Wensheng Li.

## Ethics Statement

This study was conducted according to the guidelines in the Declaration of Helsinki and all procedures involving human subjects/patients. This study was approved by the ethics committee of the Shaanxi Provincial People's Hospital (No. 2023R057). Because of the retrospective nature of the study, patient consent for inclusion was waived.

## Supporting information


**Figure S1.** MPO gene map (A) MPO partial gene sequence; (B) MPO‐463 locus genotype: GG genotype (wild type, single peak indicated by arrow); (C) MPO‐463 locus genotype: GA genotype (heterozygous mutant, double peak indicated by arrow); (D) MPO‐463 locus genotype: AA genotype (pure mutant, single peak indicated by arrow).

**Table S1.** Primer sequences.
**Table S2.** qRT‐PCR amplification reaction system.
**Table S3.** Comparison of CD5 antigen loss between Kikuchi disease and reactive hyperplastic lymph nodes.
**Table S4.** CD5 antigen loss in Kikuchi’s disease by histologic subtype.
**Table S5.** Comparison of the expression rates of MPO immunohistochemical staining in each group.
**Table S6.** Comparison of MPO mRNA expression in Kikuchi disease group and control group.
**Table S7.** Correlation between *MPO* mRNA expression and histologic subtypes in Kikuchi’s disease.
**Table S8.** Correlation between MPO protein expression (IHC scores) and *MPO* mRNA expression (ΔCt values) in Kikuchi disease and control groups.
**Table S9.** Comparison of Kikuchi’s disease with reactive hyperplastic lymph nodes for mutations at the MPO‐463 locus.
**Table S10.** Hardy–Weinberg equilibrium test for MPO‐463G/A genotype distributions in kikuchi disease and control groups.
**Table S11.** Relationship between MPO‐463G/A gene polymorphisms and *MPO* mRNA expression levels.

## Data Availability

The data that support the findings of this study are available from the corresponding author upon reasonable request.
