# Supplementary material for: Unveiling the Molecular Landscape of MPO in Kikuchi's Disease: Protein Expression, mRNA Levels, and Genetic Polymorphisms
Source: Int J Lab Hematol. 2025 Jul 19;47(5):898–905. doi: 10.1111/ijlh.14509 (PMC12426808; doi:10.1111/ijlh.14509)
Supplement: Supplementary file 1 — Figure S1. MPO gene map (A) MPO partial gene sequence; (B) MPO‐463 locus genotype: GG genotype (wild type, single peak indicated by arrow); (C) MPO‐463 locus genotype: GA genotype (heterozygous mutant, double peak indicated by arrow); (D) MPO‐463 locus genotype: AA genotype (pure mutant, single peak indicated by arrow). [file IJLH-47-898-s001.docx]

**Figure S1**. MPO gene map A. MPO partial gene sequence; B. MPO-463 locus genotype: GG genotype (wild type, single peak indicated by arrow); C. MPO-463 locus genotype: GA genotype (heterozygous mutant, double peak indicated by arrow); D. MPO-463 locus genotype: AA genotype (pure mutant, single peak indicated by arrow)
