# Supplementary material for: Unveiling the Molecular Landscape of MPO in Kikuchi's Disease: Protein Expression, mRNA Levels, and Genetic Polymorphisms
Source: Int J Lab Hematol. 2025 Jul 19;47(5):898–905. doi: 10.1111/ijlh.14509 (PMC12426808; doi:10.1111/ijlh.14509)
Supplement: Supplementary file 2 — Table S1. Primer sequences. Table S2. qRT‐PCR amplification reaction system. Table S3. Comparison of CD5 antigen loss between Kikuchi disease and reactive hyperplastic lymph nodes. Table S4. CD5 antigen loss in Kikuchi’s disease by histologic subtype. Table S5. Comparison of the expression rates of MPO immunohistochemical staining in each group. Table S6. Comparison of MPO mRNA expression in Kikuchi disease group and control group. Table S7. Correlation between MPO mRNA expression and histologic subtypes in Kikuchi’s disease. Table S8. Correlation between MPO protein expression (IHC scores) and MPO mRNA expression (ΔCt values) in Kikuchi disease and control groups. Table S9. Comparison of Kikuchi’s disease with reactive hyperplastic lymph nodes for mutations at the MPO‐463 locus. Table S10. Hardy–Weinberg equilibrium test for MPO‐463G/A genotype distributions in kikuchi disease and control groups. Table S11. Relationship between MPO‐463G/A gene polymorphisms and MPO mRNA expression levels. [file IJLH-47-898-s002.docx]

**Table S1.** Primer sequences

| Primer | Primer sequences |
| --- | --- |
| Forward primer - MPO | 5'-CGGTATAGGCACACAATGGTGAG-3' |
| Reverse primer - MPO | 5'-CAATGGTTCAAGCGATTCTTC-3' |
| Forward primer - βactin | 5'-AGCGAGCATCCCCCAAAGTT-3' |
| Reverse primer- βactin | 5'-GGGCACGAAGGCTCATCATT-3' |

**Table S2.** qRT-PCR amplification reaction system

| Reagent | Amount used (single well) |
| --- | --- |
| TB Green Premix Ex Taq II（2 X） | 10 μl |
| MPO/β-actin Forward Primer（10 μM） | 0.8 μl |
| MPO/β-actin Reverse Primer（10 μM） | 0.8 μl |
| DNA Templates | 2.0 μl |
| RNase Free ddH_2_O | 6.4 μl |
| Total | 20 μl |

**Table S3.** Comparison of CD5 Antigen Loss Between Kikuchi Disease and Reactive Hyperplastic Lymph Nodes

| Group（n，%） | Total cases | CD5 loss [n (%)] | CD5 retained [n (%)] | *Ρ* Value |
| --- | --- | --- | --- | --- |
| Kikuchi disease | 43 | 29（67.4%） | 14（32.6%） | ＜0.001^a^ |
| Reactive hyperplastic lymph nodes | 10 | 0（0） | 10（100） |  |

Note: *Ρ* ＜ 0.05 is statistically significant

**Table S4**. CD5 antigen loss in Kikuchi’s disease by histologic subtype

| Histologic Subtype | Total cases (n) | CD5 loss [n (%)] | CD5 retained [n (%)] | *P* value |  |
| --- | --- | --- | --- | --- | --- |
| PT | 17 | 7 (41.2) | 10 (58.8) | 0.005 |  |
| NT | 23 | 20 (87.0) | 3 (13.0) |  |  |
| XT | 3 | 2 (66.7) | 1 (33.3) |  |  |

Note: *Ρ* ＜ 0.05 is statistically significant.

**Table S5**. Comparison of the expression rates of MPO immunohistochemical staining in each group

| Group（n，%） | Total cases | Positive | Negative | *Ρ* Value |
| --- | --- | --- | --- | --- |
| Kikuchi disease | 43 | 43（100） | 0（0） | - |
| Reactive hyperplastic lymph nodes | 10 | 0（0） | 10（100） | ＜0.001^a^ |
| Granulomatous inflammation | 10 | 0（0） | 10（100） | ＜0.001^b^ |

Note: *Ρ* ＜ 0.05 is statistically significant. a: Kikuchi disease vs. Reactive hyperplastic lymph nodes; b: Kikuchi disease vs. Granulomatous inflammation.

**Table S6**. Comparison of MPO mRNA expression in Kikuchi disease group and control group

| Group | MPO mRNA（ΔCt）‾x±s | t Value | *Ρ* Value |
| --- | --- | --- | --- |
| Kikuchi disease | 6.31±2.96 | - | - |
| Myeloid sarcoma | 4.76±1.77 | 2.01 | 0.062^a^ |
| Granulomatous inflammation | 8.49±2.49 | 2.78 | 0.010^b^ |
| Reactive hyperplastic lymph nodes | 9.64±1.95 | 5.79 | ＜ 0.001^c^ |

Note: *Ρ* ＜ 0.05 is statistically significant. a: Kikuchi disease vs. Myeloid sarcoma; b: Kikuchi disease vs. Granulomatous inflammation; c: Kikuchi disease vs. Reactive hyperplastic lymph nodes.

**Table S7.** Correlation between *MPO* mRNA expression and histologic subtypes in Kikuchi’s disease

| Histologic Subtype | *MPO* mRNA（ΔCt）‾x±s | | F value | *Ρ* value |
| --- | --- | --- | --- | --- |
| PT | 5.29±3.17 | 1.50 | | 0.235 |
| NT | 6.76±2.56 |  |  |  |
| XT | 7.24±3.82 |  |  |  |

Note: *Ρ* ＜ 0.05 is statistically significant.

**Table S8.** Correlation between MPO protein expression (IHC scores) and *MPO* mRNA expression (ΔCt values) in Kikuchi disease and control groups

| Group | ‾x±s | r Value | *Ρ* Value |
| --- | --- | --- | --- |
| MPO Protein | 7.16±3.20 | -0.525 | ＜ 0.001 |
| MPO（ΔCt） | 6.31±2.96 |  |  |

Note: *Ρ* ＜ 0.05 is statistically significant.

**Table S9.** Comparison of Kikuchi's disease with reactive hyperplastic lymph nodes for mutations at the MPO-463 locus

| Genotype | Kikuchi disease（n，%） | Reactive hyperplastic lymph nodes（n，%） | *Ρ* Value |
| --- | --- | --- | --- |
| Wild type（GG） | 40（93.0） | 21（70） | 0.015 |
| Mutant type（GA+AA） | 3（7.0） | 9（30） |  |

Note: *Ρ* ＜ 0.05 is statistically significant.

**Table S10.**Hardy–Weinberg Equilibrium Test for MPO-463G/A Genotype Distributions in Kikuchi Disease and Control Groups

| Group | Genotype | Observed [n (%)] | Expected [n (%)] |  *P* Value |
| --- | --- | --- | --- | --- |
| Kikuchi disease | GG | 40 (93.0) | 39.09 (90.9) | 0.676 |
|  | GA | 2 (4.7) | 3.81 (8.9) |  |
|  | AA | 1 (2.3) | 0.09 (0.2) |  |
| Reactive hyperplasia | GG | 21 (84.0) | 20.92 (83.7) | >0.999 |
|  | GA | 8 (16.0) | 8.26 (16.5) |  |
|  | AA | 1 (4.0) | 0.82 (3.3) |  |

Note: Expected frequencies were calculated based on observed allele frequencies using the Hardy–Weinberg equilibrium formula (p² + 2pq + q² = 1). *P* values were calculated using the chi-square test. A *P* value > 0.05 indicates no significant deviation from Hardy–Weinberg equilibrium, suggesting the population is genetically representative.

**Table S11.** Relationship between MPO-463G/A gene polymorphisms and *MPO* mRNA expression levels

| Genotype | Kikuchi disease（ΔCt）‾x±s | t Value | *Ρ* Value |
| --- | --- | --- | --- |
| GG | 6.30±2.93 | 0.178 | 0.874 |
| GA+AA | 5.94±3.43 |  |  |

Note: *Ρ* ＜ 0.05 is statistically significant.
